# A versatile plasmid system for reconstitution and analysis of mammalian ubiquitination cascades in yeast

**DOI:** 10.15698/mic2018.03.620

**Published:** 2017-12-05

**Authors:** Rossella Avagliano Trezza, Janny van den Burg, Nico van den Oever, Ben Distel

**Affiliations:** 1 Department of Medical Biochemistry, Academic Medical Center, University of Amsterdam, Amsterdam, The Netherlands.; 2 Department of Neuroscience, Erasmus Medical Center, Rotterdam, The Netherlands.

**Keywords:** ubiquitination, sumoylation, modular vectors, inducible expression, yeast, Saccharomyces cerevisiae

## Abstract

Ubiquitination is a posttranslational protein modification that regulates most aspects of cellular life. The sheer number of ubiquitination enzymes that are present in a mammalian cell, over 700 in total, has thus far hampered the analysis of distinct protein ubiquitination cascades in a cellular context. To overcome this complexity we have developed a versatile vector system that allows the reconstitution of specific ubiquitination cascades in the model eukaryote *Saccharomyces cerevisae* (baker’s yeast). The vector system consists of 32 modular yeast shuttle plasmids allowing inducible or constitutive expression of up to four proteins of interest in a single cell. To demonstrate the validity of the system, we show that co-expression in yeast of the mammalian HECT type E3 ubiquitin ligase E6AP (E6-Associated Protein) and a model substrate faithfully recapitulates E6AP-dependent substrate ubiquitination and degradation. In addition, we show that the endogenous sumoylation pathway of *S. cerevisiae* can specifically sumoylate mouse PML (Promyelocytic leukemia protein). In conclusion, the yeast vector system described in this paper provides a versatile tool to study complex post-translational modifications in a cellular setting.

## INTRODUCTION

The process of ubiquitination, involving the covalent conjugation of the small protein ubiquitin (Ub) to a substrate protein, is one of the most finely tuned mechanisms of post-translational modification in eukaryotic biology 1. Ubiquitination is accomplished through an elaborate enzymatic cascade involving the concerted action of three distinct types of enzymes: E1 Ub-activating, E2 Ub-conjugating and E3 Ub ligase. First, Ub is activated by one of two cellular E1 enzymes, then transferred to ~40 E2 enzymes that interact with more than 600 E3 ligases. The substrate specificity is determined in the last step of the cascade, catalysed by the E3, in which Ub is covalently attached to a lysine residue of a specific target protein 2. The two main groups of E3 ligases are HECT (Homologous to E6-AP C-terminus) and RING (Really interesting new gene) ligases. The HECT E3s contain a conserved active site cysteine that acts as an acceptor for Ub from E2 conjugating enzymes. The Ub is then transferred onto a Lysine residue of the target protein. RING E3s ligases on the other hand do not have an active site cysteine, but function as a scaffold that enhances the transfer of Ub from the E2 active site directly onto the substrate 3.

Ub itself contains seven lysine residues and a N-terminal NH_2_ group, each of which can serve as a substrate of ubiquitination, resulting in the formation of Ub chains consisting of multiple Ub moieties. This ability of Ub to form chains of at least eight different linkages adds to the complexity of the ubiquitination system. Indeed, the fate of the ubiquitinated substrate is, to a large extent, determined by the length and type of linkage between Ub moieties 4. For example, chains linked via Lys63 (K63) are implicated in DNA repair, signaling and endocytosis 5. On the other hand, substrates that receive poly-ubiquitin chains linked via Lys48 (K48) or Lys11 (K11) are targeted for degradation by the 26S proteasome 6,7, a multi-protein complex responsible for proteolysis and ubiquitin recycling. The 26S proteasome is a conserved proteolytic machinery present in both the nucleus and the cytosol of eukaryotic cells 8. Proteasomes are also present in some bacterial species where they are involved in the degradation of pupylated proteins 9, a type of post-translational modification that is functionally analogous to ubiquitination, but chemically distinct 10.

The analysis of specific ubiquitination cascades in a cellular setting is a major challenge given that mammalian cells express more than 700 ubiquitination enzymes, which often have overlapping substrate specificities 11,12. To reduce complexity, cell-free approaches have been developed that utilize reconstituted ubiquitination cascades. However, these cell-free assays require extensive protein purification of the respective ubiquitination enzymes as well as their substrate. A bacterial ubiquitination system has recently been developed that allows the analysis of specific ubiquitination cascades in a cellular context 13. The intrinsic differences of proteasomes in bacteria 9, however, limit the analysis of ubiquitinated substrates. To overcome these limitations we have established a cellular system for reconstitution of mammalian E3 ubiquitin ligase-dependent substrate modification and degradation. This system employs the model eukaryote *Saccharomyces cerevisiae* (baker’s yeast) as a host for co-expression of E2, E3 and substrate of a specific mammalian ubiquitination cascade, while the yeast cell provides the ubiquitin and the E1 enzyme. Yeast shuttle plasmids were developed that allow expression and detection of up to four proteins of interest in a single yeast cell. Gene expression is regulated by three different types of promoters: galactose inducible GAL1 promoter (available in three different strengths), the CuSO_4 _inducible CUP promoter or the strong constitutive TEF promoter. To improve stability and detection of poly-ubiquitinated targets, a strain was constructed that lacks the ABC transporter *PDR5* rendering the cells more susceptible to the proteasome inhibitor MG132 14.

Here we demonstrate the validity of our system by showing specific ubiquitination and proteasomal degradation of an established target of E6AP, a HECT type E3 ligase that is deficient in patients with Angelman syndrome 15,16. In addition, we demonstrate that the system can also be used to study other types of post-translational modifications by showing sumoylation of the mouse PML protein in yeast.

## RESULTS AND DISCUSSION

### Plasmid construction

**Figure 1:**
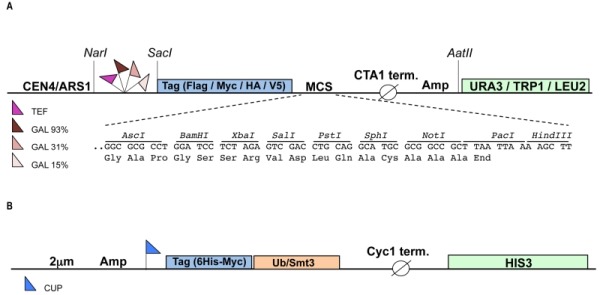
FIGURE 1: Schematic representation of the constructed vectors. The pRA vectors **(A)** are yeast centromeric shuttle plasmids that carry an auxotrophic marker (*URA3*, *TRP1* or *LEU2* gene) for selection in yeast and the ampicillin (Amp) resistance gene for selection in *E. coli. *The gene of interest can be cloned into the multiple cloning site (MCS) under control of either the inducible GAL1 promoter (3 different strengths) or the constitutive TEF promoter. The expressed protein will bear any of the four (Flag, Myc, HA or V5) N-terminal epitope tags allowing easy detection. All restriction sites indicated are unique. **(B)** High copy (2 μm) yeast shuttle plasmids carrying the *HIS3* auxotrophic marker and expressing either 6xHis-Myc tagged ubiquitin (Ub) or SUMO (Smt3) under control of the copper inducible promoter (CUP). CTA1 and Cyc1 term (inator), transcription termination sequences derived from the yeast Catalase A and Cytochrome c1 gene, respectively.

To enable study of mammalian ubiquitination cascades in yeast we constructed a modular set of plasmids (called pRA) that allow for: a) inducible or constitutive expression of the enzymes of the ubiquitination cascade and substrates, and b) detection of the gene products bearing N-terminal epitope tags. The plasmid backbone for the vector system is derived from the YCplac series of single copy yeast shuttle plasmids that carry the *URA3*, *TRP1* or *LEU2* auxotrophic selection markers 17. To induce expression of the gene of interest we made use of the tightly controlled GAL1 promoter 18. The GAL1 promoter is actively repressed by glucose and changing the carbon source from glucose to galactose induces protein expression. The use of an inducible promoter can facilitate the expression of toxic proteins 19. The GAL1 promoter exists in three different versions, "strong" (93% activity, contains four Gal4p Upstream Activating Sequences [UAS], "medium" (31% activity, three Gal4p UASs) and "weak" (15% activity, two Gal4p UASs), allowing further fine-tuning of gene expression levels. In order to facilitate constitutive expression we employed the strong TEF promoter 20. To enable protein detection four distinct N-terminal epitope tags, HA, V5, Flag and Myc were inserted in each plasmid. Finally, each plasmid harbours a new multiple cloning site (MCS) with unique restriction sites for facile cloning of the gene of interest. The pRA vector system currently consists of 32 plasmids each of which harbours a different combination of promoter, tag and auxotrophic marker (see also Table S1), but this set can be extended up to 48 by including all available GAL1 promoter variants (Fig 1). The advantage of pRA vector system compared to previously published collections of yeast expression vectors 21,22,23 is that different N-terminal epitope tags are included in the vector allowing simultaneous expression and detection of multiple genes of interest in a single yeast cell. To facilitate the detection and purification of ubiquitinated (or sumoylated) substrates two separate high copy (2 μm) plasmids were constructed that express either 6xHis-Myc tagged ubiquitin or 6xHis-Myc tagged SUMO (Smt3) under the control of the copper inducible promoter CUP1 24. These two plasmids harbor the *HIS3* auxotrophic marker and each can be combined with up to three pRA plasmids, bringing the total number of genes expressed in a single yeast cell to four.

### Galactose inducible expression

**Figure 2:**
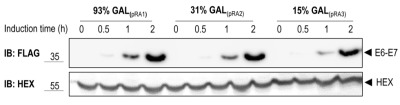
FIGURE 2: Galactose inducible expression of E6-E7. Yeast strain yRA2 transformed with E6-E7 cloned in pRA1 (Flag tag, *URA3* marker, 93%-GAL1 promoter), pRA2 (Flag tag, *URA3* marker, 31%-GAL1 promoter) and pRA3 (Flag tag, *URA3 *marker, 15%-GAL1 promoter), was induced for the indicated time points by the addition of 2% galactose. Cells were lysed and equivalent amounts of protein extract were analyzed by SDS-PAGE and anti-Flag immunoblotting. Hexokinase is used as a loading control.

To test the functionality of the GAL1 promoter plasmids we cloned E6-E7, one of the canonical targets of the HECT E3 ligase E6AP, in three different vectors harboring the 93%-, 31%- and 15%-GAL1 promoter, respectively (see Material and methods). The E6-E7 fusion protein consists of the entire human papillomavirus (HPV)-16 E7 fused to HPV-16 E6 25. E6-E7 was tagged at its N-terminus with the Flag tag. Constructs were transformed into yeast yRA2, a derivative of *S. cerevisiae* strain BY4741 in which the *TRP1* gene was knocked out (see Material and methods). Transformants were first cultured in medium containing 2% glucose, then shifted to a medium with 2% raffinose and 0.1% glucose, and finally inoculated in medium containing 2% galactose to induce expression of E6-E7 for the indicated time points. As shown in Figure 2, E6-E7 was undetectable in total lysates of cells grown in 2% raffinose/0.1% glucose medium (t=0), indicating tight glucose repression of E6-E7 expression. Within 30 min after the shift to galactose-containing medium E6-E7 could be detected in cells expressing the gene from the strongest version of the GAL1 promoter, the 93%-GAL1. All three promoter-versions showed a gradual increase of E6-E7 expression with time, reaching the highest levels with the 93%-GAL1 promoter. Taken together, these data show that by using different versions of the GAL1 promoter protein expressions levels can be modulated.

### E6AP-dependent ubiquitination of E6-E7

**Figure 3:**
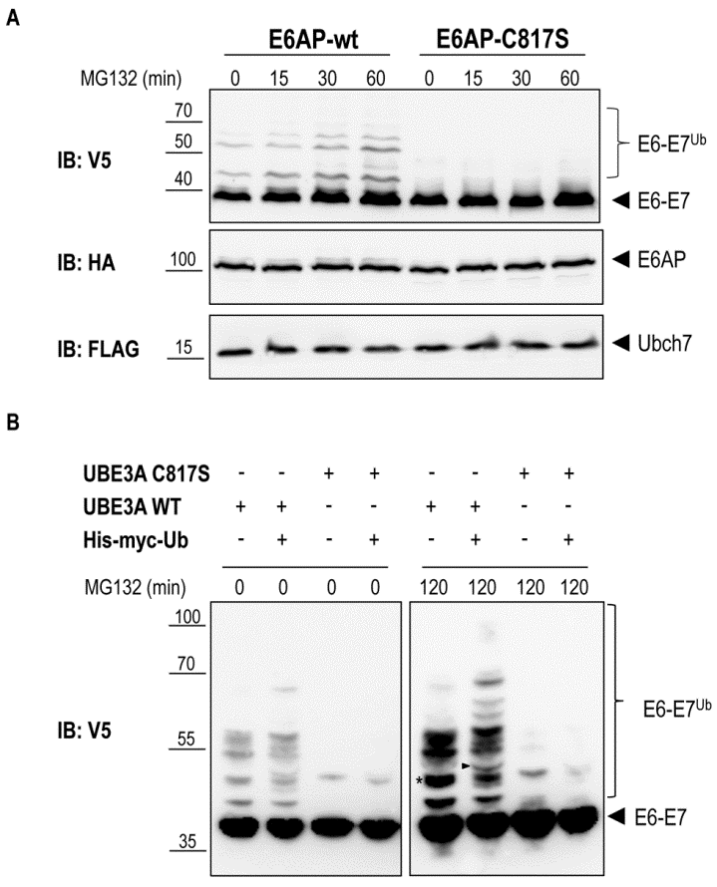
FIGURE 3: E6AP-dependent ubiquitination of E6-E7. **(A)** Yeast strain yRA2 was transformed with E6AP (wild type or catalytically inactive mutant) cloned in pRA306 (HA tag, *LEU2 *marker, TEF promoter), UBCH7 cloned in pRA300 (Flag tag, *URA3* marker, TEF promoter) and E6-E7 cloned in pRA20 (V5 tag, TRP1 marker, 93%-GAL1 promoter). Following 1 hour of galactose induction, cells were treated with 75 μM MG132 for the indicated time points. Cells were lysed and equivalent amounts of protein extract were analyzed by SDS-PAGE and immunoblotting using the indicated antibodies. **(B)** Yeast strain yRA2 was transformed with E6AP (wild type or catalytically inactive mutant) cloned in pRA306 (HA tag, *LEU2 *marker, TEF promoter), E6-E7 cloned in pRA20 (V5 tag, TRP1 marker, 93%-GAL1 promoter) and 6xHis-Myc-Ub (Myc tag, *HIS3* marker, CUP promoter) or empty p423 vector. Expression of 6xHis-Myc-Ub was induced by addition of CuSO_4 _(100 μM). Following 1 hour of galactose induction, cells were treated with 50 μM MG132 for 120 min. Cells were lysed and equivalent amounts of protein extract were analyzed by SDS-PAGE and immunoblotting using anti-V5 antibody. The band marked with an asterisk (*) is an example of an ubiquitinated E6-E7 band that in cells overexpressing 6xHis-Myc-Ub, generates additionally slower migrating species (arrow head) due to the incorporation of the 6xHis-Myc-tagged ubiquitin.

Previous work has shown that the E6-E7 fusion protein is efficiently ubiquitinated by E6AP in a cell-free system 25. We therefore set out to determine whether E6AP is capable of specifically ubiquitinating the E6-E7 fusion protein in the heterologous yeast system. To reconstitute this specific ubiquitination cascade in yeast, mouse E6AP (wild-type and the catalytically inactive mutant, E6AP-C817S) and its cognate E2, mouse UBCH7, were cloned under control of the constitutive TEF promoter, each bearing different tags (HA for E6AP and Flag for UBCH7). The yRA2 strain, in which the plasma-membrane ABC transporter Pdr5 was deleted by homologous recombination, was co-transformed with E6-E7 (V5 tag, 93%-GAL1 promoter), E6AP (wt or mutant) and either UBCH7 or an empty plasmid. Transformants were pre-cultured on 2% raffinose/0.1% glucose and shifted to 2% galactose to induce the expression of E6-E7. After 1 hour of galactose induction, 75 µM MG132 was added to inhibit proteasomal activity and allow accumulation of ubiquitinated proteins 26. Immunoblot analysis of yeast total lysates revealed that, in addition to the band corresponding to the unmodified E6-E7 fusion protein, slower migrating bands accumulated in cells expressing wild type E6AP (Figure 3A). The fact that these bands are absent in cells co-expressing the catalytically inactive E6AP (E6AP-C817S) suggests that E6-E7 can be ubiquitinated in yeast in an E6AP-dependent manner. To unambiguously show that these slower migrating spe-cies represent (poly)-ubiquitinated forms of E6-E7 the experiment was repeated with cells co-transformed with 6xHis-Myc-tagged ubiquitin (6xHis-Myc-Ub, driven by the CUP1 promoter) or empty plasmid. Efficient conjugation of 6xHis-Myc-Ub to proteins was verified by anti-myc immunoblotting (Figure S1). Conjugation of the recombinant 6xHis-Myc-Ub to a protein will result in a slower electrophoretic mobility of the ubiquitinated protein compared to a protein modified with wild-type (endogenous) ubiquitin due to the addition of the 6xHis-Myc tag on ubiquitin. As shown in Figure 3B (right panel) the slower migrating E6-E7 bands observed in cells expressing the catalytically active E6AP shifted to an apparent higher molecular weight upon overexpression of 6xHis-Myc-Ub. This data confirm that these slower migrating bands are indeed the result of E6AP-dependent ubiquitination of E6-E7. Notably, in the absence of UBCH7, similar levels of E6-E7 ubiquitination were observed indicating that one of the endogenous yeast E2 enzymes 27 can functionally interact with E6AP to ubiquitinate its target (Figure S2). This data support the notion that for the analysis of a specific mammalian ubiquitination cascade in yeast, expression of the E3 ligase and its target may be sufficent, a major advantage in cases where not all components of the specific ubiquitination cascade are known.

### Degradation of E6-E7

**Figure 4:**
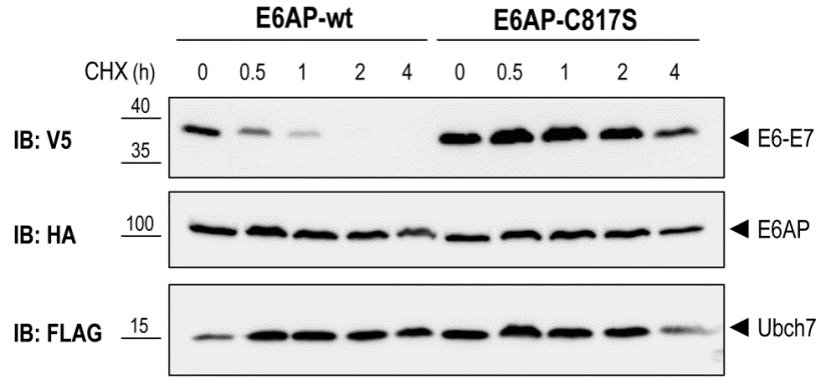
FIGURE 4: E6AP-dependent degradation of E6-E7. Yeast strain yRA2 was transformed with E6AP, UBCH7 and E6-E7 as described in the legend to Figure 3. Following 1 hour of galactose induction, cells were treated with 50 μg/ml CHX for the indicated time points. Cells were lysed and equivalent amounts of protein extract were analyzed by SDS-PAGE and immunoblotting using the indicated antibodies.

To test if E6AP-dependent poly-ubiquitination of E6-E7 leads to proteasome-mediated degradation of the fusion protein in the reconstituted yeast system, we performed a cycloheximide (CHX) chase experiment. Yeast cells were co-transformed with E6-E7, UBCH7, and E6AP-wt or E6AP-C817S. After galactose induction, protein synthesis was inhibited by treating the cells with 50 μg/ml CHX for the indicated times (Figure 4). In cells expressing E6AP-wt, E6-E7 rapidly disappeared upon addition of CHX. In contrast, no appreciable reduction of E6-E7 levels was observed in cells co-transformed with the catalytically inactive E6AP (E6AP-C817S). These data indicate that E6AP-catalyzed ubiquitination of E6-E7 in yeast results in rapid protein degradation, a process most likely mediated by the 26S proteasome. Together, we show that a complex cellular mechanism such as the ubiquitination and degradation of a substrate via the ubiquitin proteasome system can be reconstituted in yeast by expressing only the specific E3 and its substrate. Such an approach is only possible in a model eukaryotic setting, such as yeast, where biological mechanisms are highly conserved.

### Sumoylation of promyelocytic leukemia protein

Another example of a biological process that is conserved between mammals and yeast is sumoylation, a post-translational modification very similar to ubiquitination 28. Sumoylation is the process by which proteins are covalently tagged with a SUMO (small ubiquitin- like modifier) moiety that shares ~20% similarity with Ub. SUMO proteins are highly conserved from yeast to mammals. In yeast, only one SUMO protein (Smt3) exists, while mammalian cells express three major SUMO isoforms (SUMO-1, SUMO-2, and SUMO-3). Sumoylation, the linkage of SUMO to a target protein through an isopeptide bond, is catalyzed by a series of enzymatic reactions similar to that of ubiquitination. First, SUMO/Smt3 is activated by an E1 SUMO-activating enzyme (SAE1/2 in mammals and UBA2 in yeast), which is followed by transfer of the activated SUMO/Smt3 to a SUMO-specific E2, Ubc9. From here, either the SUMO/Smt3 is directly transferred to a lysine side-chain of the target protein or the transfer is indirectly and requires an SUMO E3 ligase. In contrast to ubiquitination, sumoylation often occurs on a consensus motif, Ψ-K-x-D/E (Ψ; hydrophobic residue, K; lysine, x; any amino acid and D/E; aspartic acid/glutamic acid) 28. The PML protein offers a well-established example of a protein that is sumoylated. PML is the key player in the generation of Nuclear Bodies (NB) involved in a myriad of processes, including cellular senescence, virus response, and apoptosis 29. PML contains three sumoylation consensus motifs each of which are targeted by sumoylation. Sumoylation of these consensus lysines is essential for the recruitment of nuclear proteins to the NB 30,31.

**Figure 5:**
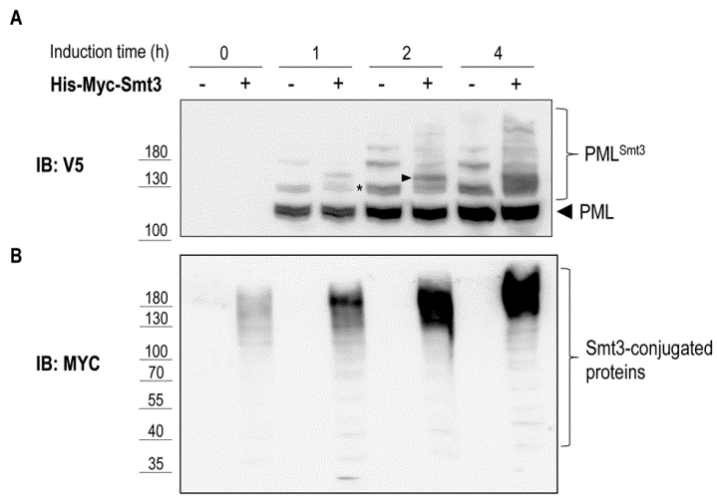
FIGURE 5: Sumoylation of mouse PML in yeast. Yeast strain yRA1 was transformed with PML cloned in pRA20 (V5 tag,* TRP1* marker, 93%-GAL1 promoter) and with a plasmid expressing 6xHis-Myc-Smt3 under control of the CUP promoter (*HIS3 *marker) or with empty vector. Expression of PML and Smt3 was induced by addition of galactose (2% [w/v]) and CuSO_4_ (100 μM), respectively. Cells were lysed and equivalent amounts of protein extract were analyzed by SDS-PAGE and anti-V5 **(A)** or anti-Myc **(B)** immunoblotting. The band marked with an asterisk (*) in panel A is an example of PML modified by a single Smt3 moiety that, in cells overexpressing 6xHis-myc-Smt3, gives rise to a slower migrating species (arrow head) due to covalent attachment of the 6xhis-Myc-tagged Smt3.

To test if PML sumoylation can be recapitulated in yeast we overexpressed V5-tagged mouse PML under the control of the 93%-GAL1 promoter in presence or absence of 6xHis-Myc-Smt3 driven by the CUP1 promoter. As shown in Figure 5A, following galactose-induction and in the absence of 6xHis-Myc-Smt3 up to three slower migrating bands appeared above the main band representing PML, suggesting targeting of all three consensus-sumoylation sites in PML. In cells overexpressing 6xHis-Myc-Smt3 these slower migrating bands were split up in two or more bands indicating successful incorporation of recombinant 6xHis-Myc-Smt3 and supporting the notion that these slower migrating species represent sumoylated forms of PML. Similar results were obtained when human PML was expressed in yeast (data not shown). This data strongly suggest that sumoylation of mouse PML can be faithfully reproduced in yeast by employing its endogenous sumoylation machinery. In line with previous observations 32 overexpression of PML resulted in an overall increase of Smt3-conjugated species, most likely representing Smt3 linked to PML and to endogenous yeast proteins (Fig. 5B). How PML stimulates Smt3 conjugation in yeast remains to be determined.

In conclusion, we show that the use of a model eukaryote, in combination with a highly versatile plasmid system that allows regulated expression of multiple heterologous proteins, can facilitate the analysis of complex (mammalian) post-translational modification cascades such as ubiquitination and sumoylation in a cellular context.

## MATERIALS AND METHODS

### Plasmid generation

The vector backbone of the pRA plasmids is pEL43 33, an YCplac33 derived yeast shuttle plasmid 17 harboring the CTA (Catalase A) promoter and CTA terminator separated by a multiple cloning site (MCS), the *URA3* auxotrophic marker and the *ARS1/CEN4* region for autonomous replication in yeast. The three different GAL1 promoters (93%, 31% and 15%) were obtained by PCR on yeast genomic DNA using primers containing a 5’ *Nar*I and a 3’ *Sac*I site, respectively (Table S2) and subsequently cloned in pEL43 digested with *Nar*I and *Sac*I, thereby replacing the CTA promoter with the GAL1 promoter sequences. Next, a new MCS was inserted in each of the three vectors between the *Pst*I and *Hind*III sites using double stranded (ds) oligonucleotides (Table S2). At this point, ds oligonucleotides encoding the four different N-terminal epitope tags (HA, V5, Flag and Myc) followed by an *Asc*I site were inserted between the *Sac*I and *BamH*I restriction sites of each plasmid. Finally, the *URA3* auxotrophic marker in these plasmids was replaced by *TRP1* or *LEU2* by cloning the *Nar*I-*Aat*II fragments encompassing promoter, tag, MCS and CTA terminator into YCplac22 (*TRP1*) and Ycplac111 (*LEU2*) digested with *Nar*I and *Aat*II. The TEF promoter was obtained by PCR from pTEF-URA 34 (generous gift of Jens Nilsen) using primers containing a 5’ *Nar*I and a 3’ *Sac*I and subsequently cloned in pRA1, 4, 7, 10 and 13-20, digested with *Nar*I and *Sac*I, thereby replacing the 93%-GAL1 promoter with the TEF promoter sequence. *Ubch7* (Ube2l3) was amplified by PCR from image clone BC106149 (clone ID 4502101) using primers containing a 5’ *Asc*I and a 3’ *Sal*I site, respectively (Table S2) and subsequently cloned in pRA300 digested with *Asc*I and *Sal*I. To obtain a higher expression of the E2 enzyme, the *CEN/ARS1* sequence in pRA58 was replaced by the 2 μm origin of replication derived from YEplac195, resulting in plasmid pRA60. *Ube3a* was amplified by PCR with primers p881 and p882 from mouse brain cDNA (generous gift of Ype Elgersma) and subsequently cloned in pRA306. *Pml* was amplified by PCR from image clone BC020990 (clone ID 4188386) using primers containing a 5’ *Asc*I and a 3’ *Sal*I site, respectively (Table S2) and subsequently cloned in pRA20. E6-E7 fusion protein was amplified by fusion PCR (to delete the internal *Sal*I site) from plasmid #95 25 (generous gift of Jon Huibregtse) using external primers containing a 5’ *Bam*HI and a 3’ *Sal*I site, respectively (S2 Table) and subsequently cloned in pRA20, generating pJB322. The *BamH*I-*Not*I fragment encompassing E6-E7 was isolated from pJB322 and cloned in plasmids pRA1, pRA2 and pRA3 digested with *BamH*I and*Not*I.

6xHis-Myc-Ub and 6xHis-Myc-Smt3 plasmids are based on p423, a multi-copy (2 μm) yeast shuttle plasmid harboring the MET25 promoter, Cyc1 terminator and *HIS3 *auxotrophic marker 23. A three fragment ligation was used to construct 6xHis-Myc-Ub as follows: the *Eco*RI-*Mlu*I fragment from pCW129 35 encompassing 6xHis-Myc-Ub and part of the Cyc1 terminator sequences and the *Sac*I-*Eco*RI fragment from pES7 (generous gift of Mike Ellison, University of Alberta) harboring the CUP1 promoter were ligated into p423 digested with *Sac*I and *Mlu*I, generating 6xHis-Myc-Ub (pBD238, Table S2). To construct 6xHis-Myc-Smt3, the Smt3 gene was amplified from yeast genomic DNA, thereby introducing a 5’ *BamH*I site and a 3’ *Kpn*I site. The *Bam*HI-*Kpn*I fragment was subsequently cloned into pCW129 digested with *Bgl*II and *Kpn*I, thereby replacing the Ub gene with Smt3. The *Bam*HI-*Mlu*I fragment of the resulting plasmid, encompassing the CUP promoter, 6xHis-Myc-Smt3 and part of the Cyc1 terminator sequences, was isolated and cloned into pBD238 cut with *Bam*HI and *Mlu*I, generating 6xHis-Myc-Smt3 (pNO3, S1 Table). A complete list of plasmids generated in this study can be found in Table S1. Plasmids will be deposited at Addgene.

### Yeast strains

Strain yRA1 was derived from *S. cerevisiae* strain BY4741 (MAT*a* *his3Δ1 leu2Δ0 met15Δ0 ura3Δ0*) by partial deletion of the *TRP1 *gene (0.51 kb deletion) using the *loxP*/Cre gene disruption and marker rescue procedure as previously described 36. This deletion keeps elements of the UAS (upstream activating sequence) required for expression of the adjacent *GAL3* gene intact thereby not affecting the kinetics of galactose induction 37. The *loxP*/Cre system was also used to delete the ABC transporter gene *PDR5* in yRA1 thereby generating yRA2.

### Yeast transformation

Transformation of *S. cerevisiae* cells was conducted as previously described using the lithium-acetate protocol 38.

### Galactose induction

On day 1, yeast colonies transformed with the desired plasmid(s) were inoculated in 3 ml of minimal medium (2% [w/v] glucose, 0.67% [w/v] Yeast Nitrogen Base [DIFCO] and amino acids as required) and incubated over night (O/N) at 28°C while shaking at 200 rpm.

On day 2, cells of the O/N culture were re-inoculated in 10 ml of minimal medium at an OD_600 _of 0.2 and grown until they reached an OD_600 _of 0.8 (approx. 7-8h). Upon reaching the desired OD value, cells were re-inoculated in 10 ml of raffinose minimal medium (2% [w/v] raffinose, 0.1% [w/v] glucose, 0.67% [w/v] Yeast Nitrogen Base [DIFCO] and amino acids as required) at a starting OD_600_ of 0.05 and grown O/N (16h) at 28°C while shaking (200 rpm).

On day 3, O/N cultures were re-inoculated at OD_600_ of 0.2 in rich raffinose medium (2% [w/v] Bacto peptone, 1% [w/v] Bacto yeast extract, 2% [w/v] raffinose, 0.1% [w/v] glucose) and grown to an OD_600_ of 0.5 (approx. 3-4h). An uninduced sample was taken before adding 2% galactose [w/v] to the cultures in order to induce protein expression. At the desired time points, samples were taken and the absorbance measured at 600 nm.

### Ubiquitination assay

For the ubiquitination assay, cultures were treated with 75 μM or 50 μM MG132 (SelleckBio) or an equivalent volume of DMSO after 1 hour of galactose induction. Samples were taken at the indicated time points and total protein lysates were prepared.

### Degradation assay

For the degradation assay, cultures were treated with 50 μg/ml cycloheximide (CHX)(Sigma) after 1 hour of galactose induction. Samples were collected at the indicated time points in ice-cold sodium azide (10 mM final concentration) to prevent undesired protein degradation.

### Protein extracts and immunoblotting 

Protein extracts were prepared by breaking the cells with glass beads and acid precipitation essentially as previously described 39. Aceton washed protein pellets were dried, re-suspended in 1X Laemmli-sample buffer (0.2 M Tris-HCl [pH 6.8], 1.5% sodium dodecyl sulfate [SDS], 10% glycerol, 1 mM EDTA, 0.004% bromophenol blue) containing 50mM DTT, heated for 10 min at 65°C and separated by SDS-PAGE. Protein transfer and detection was done essentially as previously described 40. The primary antibodies used included anti-HA rat monoclonal peroxidase-conjugated antibody (3F10; Roche), anti-Myc mouse monoclonal antibody (Cell Signaling CST2276), anti-myc-HRP (Thermo Fisher R951-25), anti-V5-HRP (Thermo Fisher R961-25), anti-Flag monoclonal antibody (M2 Sigma) and anti-Hexokinase polyclonal antibody.

## SUPPLEMENTAL MATERIAL

Click here for supplemental data file.

All supplemental data for this article are also available online at http://microbialcell.com/researcharticles/a-versatile-plasmid-system-for-reconstitution-and-analysis-of-mammalian-ubiquitination-cascades-in-yeast/.
