# Supplementary material for: A versatile plasmid system for reconstitution and analysis of mammalian ubiquitination cascades in yeast
Source: Microb Cell. 2017 Dec 5;5(3):150–7. doi: 10.15698/mic2018.03.620 (PMC5826702; doi:10.15698/mic2018.03.620)
Supplement: Supplementary file 1 [file mic-05-150-s01.pdf]

SUPPLEMENTAL DATA

Table S1. Plasmids generated in this study

Table S2. Primers used in this study

Figure S1. Efficient incorporation of 6xHis-Myc-Ub

Figure S2. E6AP-dependent ubiquitination of E6-E7 in the absence of mouse UbcH7

Table S1 Plasmids generated in this study

Name	Promoter	Tag	Marker	Insert
pRA1	93%-GAL1	Flag	URA3	-
pRA2	31%- GAL1	Flag	URA3	-
pRA3	15%- GAL1	Flag	URA3	-
pRA4	93%- GAL1	Myc	URA3	-
pRA5	31%- GAL1	Myc	URA3	-
pRA6	15%- GAL1	Myc	URA3	-
pRA7	93%- GAL1	HA	URA3	-
pRA8	31%- GAL1	HA	URA3	-
pRA9	15%- GAL1	HA	URA3	-
pRA10	93%- GAL1	V5	URA3	-
pRA11	31%- GAL1	V5	URA3	-
pRA12	15%- GAL1	V5	URA3	-
pRA13	93%- GAL1	Flag	LEU2	-
pRA14	93%- GAL1	Myc	LEU2	-
pRA15	93%- GAL1	HA	LEU2	-
pRA16	93%- GAL1	V5	LEU2	-
pRA17	93%- GAL1	Flag	TRP1	-
pRA18	93%- GAL1	Myc	TRP1	-
pRA19	93%- GAL1	HA	TRP1	-
pRA20	93%- GAL1	V5	TRP1	-
pRA36	93%- GAL1	V5	TRP1	PML
pRA53	TEF	HA	LEU2	E6AP
pRA58	TEF	Flag	URA3	UBCH7
pRA60	TEF	Flag	URA3	UBCH7 (2μm)
pRA62	TEF	HA	LEU2	E6AP-C817S
pRA300	TEF	Flag	URA3	-
pRA301	TEF	Myc	URA3	-
pRA302	TEF	HA	URA3	-
pRA303	TEF	V5	URA3	-
pRA304	TEF	Flag	LEU2	-
pRA305	TEF	Myc	LEU2	-
pRA306	TEF	HA	LEU2	-
pRA307	TEF	V5	LEU2	-
bloemenpRA308	TEF	Flag	TRP1	-
pRA309	TEF	Myc	TRP1	-
pRA310	TEF	HA	TRP1	-
pRA311	TEF	V5	TRP1	-
pBD238	CUP	6xHis-Myc	HIS3	Ubiquitin
pNO3	CUP	6xHis-Myc	HIS3	Smt3
pJB322	93%- GAL1	V5	TRP1	E6-E7
pRA315	93%-GAL1	Flag	URA3	E6-E7
pRA316	31%- GAL1	Flag	URA3	E6-E7
pRA317	15%- GAL1	Flag	URA3	E6-E7

Table S2 Primers used in this study

Name	Primer Sequence 5'-3'	Purpose
RA-A	GGCATGCGCGGCCGCTTAATTAAA	NotI and PacI insertion Fw
RA-B	AGCTTTTAATTAAGCGGCCGCGCATGCCTGCA	NotI and PacI insertion Rv
RA-G	AAAGGCGCCAACTGCTCATTGCTATATTGAAG	93%-GAL1 PCR Fw
RA-H	AAAGGCGCCAGCGGGCGACAGCCCTCC	31%-GAL1PCR Fw
RA-I	AAAGGCGCCGACGGAAGACTCTCCTCCG	15%-GAL1PCR Fw
RA-L	AAAGAGCTCCTTGACGTTAAAGTATAGAG	93%-, 31%-, 15%- GAL1 PCR Rv
RA-1	TACGTACAATCTTGATCCGGAGCTTTTCTTTTTTTGCCGATTAAGAAT TCGCATAGGCCACTAGTGGATC	TRP1 KO cassette Fw
RA-2	CACCAACATTTTCTGGCGTCAGTCCACCAGCTAACATAAAATGTAAGC TTCAGCTGAAGCTTCGTACGC	TRP1 KO cassette Rv
RA-C	CAAAATGGCTGATTATAAAGACGATGACGATAAATCTGGTGCTTCTGG CGCGCCTG	FLAG tag Fw
RA-D	GATCCAGGCGCGCCAGAAGCACCAGATTTATCGTCATCGTCTTTATAA TCAGCCATTTTGAGCT	FLAG tag Rv
RA-5.1	CAAAATGGCTGAACAAAAATTGATTTCTGAAGAGGATTTGTCTGGTGC TTCTGG	MYC tag Fw
RA-6.1	CGCGCCAGAAGCACCAGACAAATCCTCTTCAGAAATCAATTTTTGTTC AGCCATTTTGAGCT	MYC tag Rv
RA-7	CAAAATGGCTTATCCATATGATGTTCCAGATTATGCTTCTGGTGCTTC TGG	HA tag Fw
RA-8	CGCGCCAGAAGCACCAGAAGCATAATCTGGAACATCATATGGATAAGC CATTTTGAGCT	HA tag Rv
RA-9	CAAAATGGCTGGTAAACCAATTCCTAATCCATTGTTAGGTTTGGATTC TACTTCTGGTGCTTCTGG	V5 tag Fw
RA-10	CGCGCCAGAAGCACCAGAAGTAGAATCCAAACCTAACAATGGATTAGG AATTGGTTTACCAGCCATTTTGAGCT	V5 tag Rv
RA-66	AAAGGCGCCCCACACACCATAGCTTCAAAAT	TEF promoter Fw
RA-67	AAAGAGCTCTTTGTAATTAAAACTTAGATTAGATTGC	TEF promoter Rv
RA-16	AAAGGCGCGCCTATGGCGGCCAGCAGGAGG	*Ubch7* Fw
RA-17	AAAGTCGACTTAGTCCACAGGTCGCTTTTC	*Ubch7* Rv
RA-14	AAAGGCGCGCCTATGGAAACTGAACCAGTTTCCG	*Pml* Fw
RA-15	AAAGTCGACCTAGGCCAGGCATCCCTTAC	*Pml* Rv
RA-199	GGATCCATGGCTTTTCAGGACCCACAGGAGCG	E6 BamHI/Nco Fw
RA-482	GCAATGTAGGTGTATCTCCATGATCGACCAGCTGGGTTTCTCTACGTGT	E6-E7 SalMUT Rv
RA-483	ACACGTAGAGAAACCCAGCTGGTCGATCATGGAGATACACCTACATTGC	E6-E7 Fw
RA-484	GCGGCCGCGTCGACTTATGGTTTCTGAGAACAGATGGG	E6-E7 Sal/Not Rv
P881	GGCGCGCCTATGAAGCGAGCAGCTGCAAAGC	cDNA PCR *Ube3a* Fw
P882	GTCGACTTACCTAATCACAACAGATTC	cDNA PCR *Ube3a* Rv

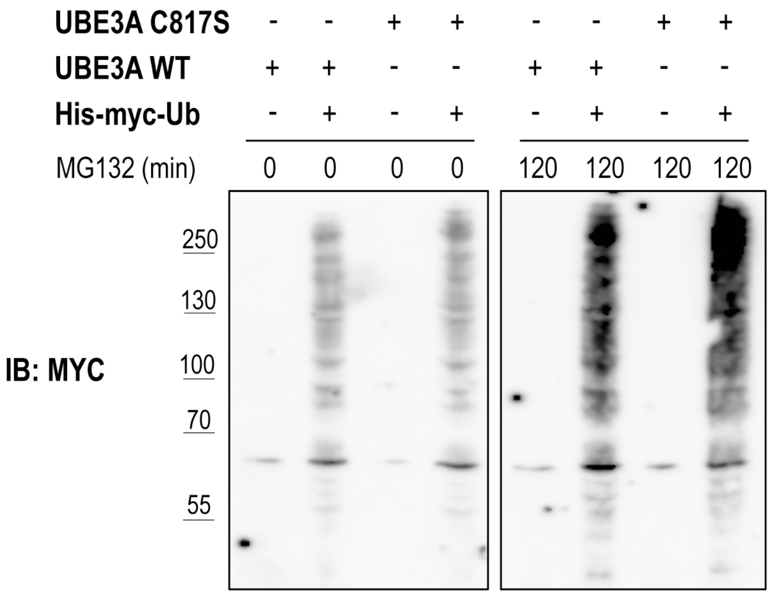

Figure S1. Efficient incorporation of 6xHis-Myc-Ub. The same yeast cell extracts employed in Figure 3B were analyzed by SDS-PAGE and immunoblotting with the anti-myc antibody.

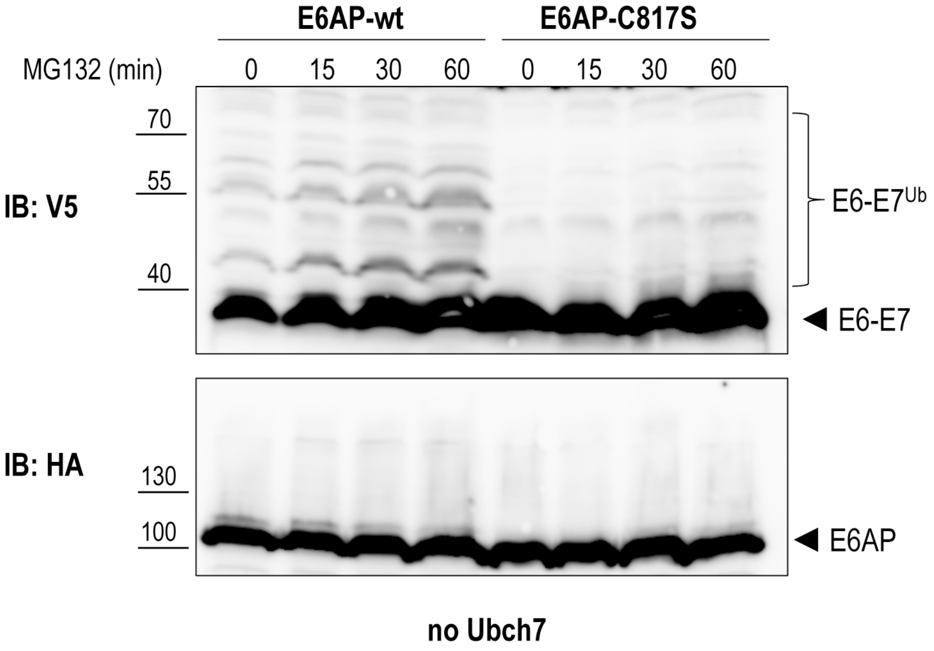

Figure S2. E6AP-dependent ubiquitination of E6-E7 in the absence of mouse UbcH7. Yeast strain yRA2 was transformed with E6AP (wild type or catalytically inactive mutant) cloned in pRA300 (HA tag, *LEU2* marker, TEF promoter) and E6-E7 cloned in pRA20 (V5 tag, *TRP1* marker, 93%-GAL1 promoter). Following 1 hour of galactose induction, cells were treated with 75 μM MG132 for the indicated time points. Cells were lysed and equivalent amounts of protein extract were analyzed by SDS-PAGE and immunoblotting using the indicated antibodies.
